# Neuropsychomotor developmental delay: conceptual map, term definitions, uses and limitations

**DOI:** 10.1016/j.rpped.2014.04.009

**Published:** 2015-03

**Authors:** Lílian de Fátima Dornelas, Neuza Maria de Castro Duarte, Lívia de Castro Magalhães

**Affiliations:** a Universidade Federal de Minas Gerais, Belo Horizonte, MG, Brazil; b Associação de Assistência à Criança Deficiente de Minas Gerais, Uberlândia, MG, Brazil

**Keywords:** Child development, Child development disorders, pervasive

## Abstract

**OBJECTIVE:**

To retrieve the origin of the term neuropsychomotor developmental delay" (NPMD), its conceptual evolution over time, and to build a conceptual map based on literature review.

**DATA SOURCE:**

A literature search was performed in the SciELO Brazil, Web of Science, Science Direct, OneFile (GALE), Pubmed (Medline), Whiley Online, and Springer databases, from January of 1940 to January of 2013, using the following keywords: NPMD delay, NPMD retardation, developmental delay, and global developmental delay. A total of 71 articles were selected, which were used to build the conceptual map of the term.

**DATA SYNTHESIS:**

Of the 71 references, 55 were international and 16 national. The terms developmental delay and global developmental delay were the most frequently used in the international literature and, in Brazil, delayed NPMD was the most often used. The term developmental delay emerged in the mid 1940s, gaining momentum in the 1990s. In Brazil, the term delayed NPMD started to be used in the 1980s, and has been frequently cited and published in the literature. Delayed development was a characteristic of 13 morbidities described in 23 references. Regarding the type of use, 19 references were found, with seven forms of use. Among the references, 34 had definitions of the term, and 16 different concepts were identified.

**CONCLUSIONS:**

Developmental delay is addressed in the international and national literature under different names, various applications, and heterogeneous concepts. Internationally, ways to improve communication between professionals have been indicated, with standardized definition of the term and use in very specific situations up to the fifth year of life, which was not found in Brazilian publications.

## Introduction

Arthur was born at a gestational age of 32 weeks, weighing 2,100 grams. At six months of life, his pediatrician referred him to physical therapy due to neuropsychomotor developmental delay (NPMD), because, according to his mother, "he could not support his head and had no tonus." The mother was told that she shouldn't worry, as it was nothing serious and, in fact, Arthur showed fast motor progress and was discharged from care. Currently, Arthur is 7 years old and has difficulty using cutlery, tying shoes, and does not perform personal hygiene tasks on his own. He cannot play ball, but loves video games. According to his mother, Arthur is a quiet child who walks, talks, sees, hears, and understands what is said to him normally, but has little initiative, is very dependent, and has difficulty in adapting to new environments and people. In school, according to the teacher, he is a shy child, but interacts with colleagues and participates in all activities requiring minimal assistance. He is learning to read and write, but is slower than peers and is inattentive. The parents are confused because the child persists with the diagnosis of NPMD, which does not qualify him to receive specialized support.

It is estimated that 200 million children worldwide, younger than five years of age, are at risk of not reaching their full development.^1^ The prevalence of developmental delay is largely unknown, but data from the World Health Organization (WHO) indicate that 10% of the population of any country consists of individuals with some type of disability, with a rate of 4.5% among those younger than five years of age.^1^

In Brazil,^2^ there has been a decrease in the prevalence of children with developmental delay, which is justified by the advances in neonatal care, the expansion of health care coverage for the child in the first year of life that occurred in recent decades in hospitals located in large cities and in the countryside, and the increase in the socioeconomic status of the population. However, these same factors have led to a paradoxical situation, as the higher survival of at-risk infants, especially the premature, is associated with increased morbidity, such as neurodevelopmental sequelae, generating new demands for pediatricians and other health professionals. 

Developmental delay is associated with several childhood conditions, from conception, pregnancy, and childbirth, due to adverse factors such as malnutrition, neurological diseases such as chronic childhood encephalopathy (cerebral palsy), and genetic factors, such as Down syndrome. The delay may also be a transient condition, which does not allow defining what the child development outcome will be and, thus, requires follow up with periodic evaluations. It can also be observed that it is not uncommon to find the term used as a diagnosis, as in the case of Arthur, without a more objective definition of what is happening with the child.

Although the term developmental delay is widely used in the area of child health, and is often employed clinically and mentioned in the literature, it is worth mentioning, as discussed by Aircadi^3^ in 1998, that the term does not appear as a chapter title or in the table of contents of most books on child neurology, or in the International Classification of Diseases - 10^th^ Revision (ICD-10) and the Diagnostic and Statistical Manual of Mental Disorders - fourth Edition (DSM-IV).

Nevertheless, what does the term developmental delay mean? According to the Dictionary of Developmental Disabilities Terminology,^4^ developmental delay is a condition in which the child is not developing and/or does not reach skills in accordance with the sequence of predetermined stages. However, this definition is not consensual and the lack of concept standardization has generated disagreement among professionals, leading to very different usage scenarios and a multitude of terms (e.g., developmental delay, neuropsychomotor developmental delay, mental retardation, delayed neuropsychomotor development, delayed global development), which do not seem to have the same meaning, although they are often used in a similar manner.^3^^,^ 5 ^,^ 6

In fact, it is a term that has baffled professionals and especially parents, as the term delay gives the idea of retardation, something that will take time to occur, or that development is slow, but the child will reach his/her final destination, i.e., that the problem is temporary and the prognosis is favorable.^7^^-^ 9 That does not always occur, as in the case of Arthur. The term has been used over the years in a generic way, not functioning as a communication tool, bringing dissatisfaction to parents, as they do not know what type of problem their child has, and causing frustration at school, because without a specific diagnosis, the child is not eligible to receive specialized educational support or assistance by the health team.

Thus, the term appears to be a byproduct of conceptual and methodological difficulties in reliably defining and measuring the skills of young children, as it can be applied indiscriminately to both a child with mild delay and one with severe impairment. An infant, for instance, who shows such delay in fine motor and language skills can receive the same label as an infant with severe motor and cognitive delay, i.e., they will be treated as if they have a homogeneous entity, both in terms of cause and prognosis.^7^^,^ 8

In practice, the physician does not always have appropriate tools, which includes valid and reliable development tests, or the support of an interdisciplinary team to assist in the diagnosis. Moreover, assessing developmental delay requires the capacity to recognize that development pathways are invariably individualized, with variations within what can be accepted as normal and abnormal,^10^^,^ 11 which implies the need for more prolonged contact to identify the context of the child's life.

Considering the frequent use and conceptual misperceptions related to the use of the term developmental delay, the objective of this study was to seek information through a literature search on the term NPMD delay, aiming to recover its conceptual origin and evolution over time, as documented in scientific articles. To organize such information, a conceptual map was constructed to provide an insight into the complexity of this terminology use.

## Method

The authors initially conducted a search on the subject at CAPES Portal using the term *atraso do desenvolvimento* (developmental delay), aiming to identify the databases that index articles on the subject. The most frequent databases were: SciELO Brazil, Web of Science, Science Direct, OneFile (GALE), Pubmed (MEDLINE), Wiley Online, and Springer. Next, searches were performed with specific terms for each database as described in Table 1. Searches and coding of data were performed by the first author.

**Table 1:** Electronic databases with the terms used and number of documents found.

Databases	Terms used	Number of documents found
Scielo Brazil	atraso do desenvolvimento; atraso do desenvolvimento neuropsicomotor; retardo do desenvolvimento neuropsicomotor, atraso do desenvolvimento global; retardo mental	498
Web of Science	developmental delay* AND child; global developmental delay*; neuropsychomotor developmental delay*; neuropsychomotor developmental retardation*; mental retardation* AND child; neurodevelopmental disabilities* AND child; developmental disorder* AND child*	7,545
Science Direct	developmental delay AND child; global developmental delay; neuropsychomotor developmental delay; neuropsychomotor developmental retardation; mental retardation AND child; neurodevelopmental disabilities AND child; developmental disorder AND child	3,672
Pubmed (MEDLINE)	developmental delay child; global developmental delay; neuropsychomotor developmental delay; neuropsychomotor developmental retardation; mental retardation child; neurodevelopmental disabilities child; developmental disorder child	8,974
Wiley Online Library	developmental delay AND child; global developmental delay AND child; neuropsychomotor developmental delay; neuropsychomotor developmental retardation; mental retardation AND child; neurodevelopmental disabilities AND child; developmental disorder AND child	3,662
Springer	developmental delay; global developmental delay; neuropsychomotor developmental delay; neuropsychomotor developmental retardation; mental retardation; neurodevelopmental disabilities; developmental disorder	4,505
OneFile (GALE)	children with developmental delays; global developmental delay; neuropsychomotor developmental delay; neuropsychomotor developmental retardation; mental retardation AND children´s; neurodevelopmental disabilities; developmental disorders	675
Total		29,531

With the objective of retrieving the origin of the term, the search strategy did not have a time limit, including from the earliest records on the subject, published in 1940, until January of 2013, resulting in 29,531 documents. Aiming to focus on more specific terms, the "filter results by topic" resource was used in each database. Through the filter, it was observed that, of the keywords used, the terms global developmental delay and developmental delay in the international literature and *atraso do desenvolvimento*, *atraso do DNPM*, and *retardo do DNPM* in national databases were the most suitable to encompass and find as many articles as possible related to the proposed objective.

**Figure 1:**
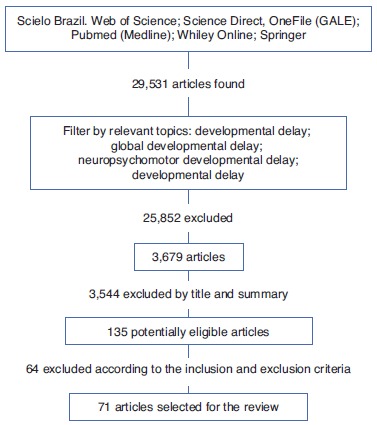
Literature search steps.

Therefore, after using the filter for these terms, 3,679 studies were selected. Subsequently, the titles and abstracts of the located articles were screened to eliminate those not related to the proposed topic. To select studies that would be read in full, as illustrated in Figure 1, the following inclusion and exclusion criteria were applied until the final sample was reached:

Inclusion criteria:

Articles published in English and Portuguese; original articles, review articles, and special articles (theoretical);

Articles that used the terms developmental delay and global developmental delay in the international literature and *atraso do DNPM* and *retardo do DNPM* in the national literature, which included at least one of the three topics below:

Population - description or indication of the type of disorder or population included in the term;

Use - description of the situation or criteria used to employ the term;

Definition - presentation of definition or concept, explaining the meaning of the term.

*Exclusion criteria:* topics that were not related to the keywords used, studies that only mentioned the term without any information, case reports, reviews, letters to the editor, testimonials, interviews, points of view, editorials, minutes of conferences, and comments of newspapers. Books and book chapters were excluded due to the difficulty in locating both the records and the material in its entirety, electronically, especially older publications. 

The selected articles were read in full for detailed data extraction, which were organized into three tables, according to the type of information obtained: the first addresses the population to which the term was applied (Table 2), the second describes how the term was used (Table 3), and the last, the term definitions (Table 4). Each table included information about the title, author and year, type of article, country, term used, and specific information about the term. In order to encompass the largest amount of information on the term use, while considering a historical perspective, it was decided not to assess the methodological quality of the articles; however, all studies met the inclusion and exclusion criteria for this review. The reference lists of the articles were assessed in an attempt to locate sources of information and theoretical data that gave rise to the term definitions and uses employed in the articles.

**Table 2:** Articles organized according to the population classified using the term neuropsychomotor developmental delay.

Nº	Title	Author, year	Type of article	Country	Term	Population
Nº	Title	Author, year	Type of article	Country	Term	Population
1-	Mental development of prematurely born children	Benton, 1940	Cross-sectional	United States	Global developmental delay	Preterm children
2-	Impact of delayed development in premature infants on mother-infant interaction: a prospective investigation	Minde et al, 1998	Longitudinal	Canada	Developmental delay	
3-	Relation between very low birth weight and developmental delay among preschool children without disabilities	Schendel et al, 1997	Cross-sectional	United States	Developmental delay	
4-	Developmental delay at 12 months in children born extremely preterm	Lando et al, 2005	Longitudinal	United States	Developmental delay	
5-	In pursuit of potential: a discussion of developmental delay, structuralization and one child’s efforts at mastery	Socor, 1981	Theoretical	United States	Developmental delay	Children with cerebral palsy
6-	Novelty responding and behavioral development in young, developmentally delayed children	Mundy et al, 1983	Cross-sectional	United States	Developmental delay	Children with chromosomal and congenital abnormalities, hydrocephalus, autism, alcoholic syndrome, and preterm children
7-	Preschool childrwen with developmental delays: nursing intervention	Steele, 1998	Descriptive	United States	Developmental delay	
8-	Benefits of early intervention for children with developmental disabilities	Majnemer, 1998	Theoretical	Canada	Global developmental delay	
9-	Neurodevelopmental delay in small babies at term: a systematic review	Arcangeli et al, 2012	Review	United Kingdom	Developmental delay	
10-	Semiology of growth restriction: diagnostic script	Marcondes, 1983	Theoretical	Brazil	Neuropsychomotor developmental delay	Children with mental retardation
11-	The etiology of developmental delay	Aircardi, 1998	Theoretical	England	Developmental delay	
12-	Radiological findings in developmental delay	Shaefer et al, 1998	Theoretical	United States	Developmental delay	
13-	Early experience and early intervention for children at risk for developmental delay and mental retardation	Ramey et al, 1999	Review	United States	Developmental delay	
14-	Diagnostic evaluation of developmental delay/mental retardation: an overview	Bataglia et al, 2003	Review	United States	Developmental delay	
15-	Peer-related social interactions of developmentally delayed young children: development and characteristics	Guranilk et al, 1984	Longitudinal	United States	Developmental delay	Children with chromosomal and congenital abnormalities and preterm children.
16-	Identifying patterns of developmental delays can help diagnose neurodevelopmental disorders	Tervo, 2006	Theoretical	United Kingdom	Global developmental delay	
17-	Differences in the memory-based searching of delayed and normally developing young children	Deloache et al, 1987	Cross-sectional	United States	Developmental delay	Children with cerebral palsy, delayed language skills and preterm children
18-	Variability in adaptive behavior in children with developmental delay	Bloom et al, 1994	Cross-sectional	United States	Developmental delay	Children with mental retardation and preterm children
19-	The negative effects of positive reinforcement in teaching children with developmental delay	Bierdeman et al, 1994	Cross-sectional	Canada	Developmental delay	
20-	Evaluation of neurodevelopmental delay in special-needs children at a university subnormal vision service	Sampaio et al, 1999	Cross-sectional	Brazil	Neuropsychomotor developmental delay	Children with sensory impairment
21-	Early rehabilitation service utilization patterns in young children with developmental delays	Majnemer et al, 2002	Longitudinal	Canada	Global developmental delay	Children with global delay, with motor delay, with language delay and autism.
22-	Global developmental delay and its relationship to cognitive	Grether, 2007	Theoretical	United States	Global developmental delay	Children with mental retardation, delayed motor development
23-	Does race influence age of diagnosis for children with developmental delay?	Mann et al, 2008	Longitudinal	United States	Developmental delay	Children with cerebral palsy, with language delay and sensory impairment

**Table 3:** Articles organized according to the use of the term neuropsychomotor developmental delay.

Nº	Title	Author, year	Type of article	Country	Term	Term use
Nº	Title	Author, year	Type of article	Country	Term	Term use
1-	The changing picture of cerebral dysfunction in early childhood	Solomons et al, 1963	Longitudinal	United States	Developmental delay	Children who do not exhibit typical development without obvious neurological signs that may indicate cerebral palsy
2-	The status at two years of low-birth-weight infants born in 1974 with birth weights of less than 1,001 gm	Pape et al, 1978	Longitudinal	Canada	Developmental delay	Children with low scores at developmental tests
3-	Relation between very low birth weight and developmental delay among preschool children without disabilities	Schendel et al, 1997	Cross-sectional	United States	Developmental delay	
4-	Screening tests and standardized assessments used to identify and characterize developmental delays	Rosenbaum, 1998	Theoretical	Canada	Developmental delay	
5-	Effects of testing context on ball skill performance in 5 year old children with or without developmental delay	Doty et al, 1999	Cross-sectional	United States	Developmental delay	
6-	Etiologic evaluation in 247 children with global developmental delay at Istanbul Turkey	Ozmen et al, 2005	Cross-sectional	Turkey	Global Developmental delay	
7-	Assessment of the neuropsychomotor development of children living in the outskirts of Porto Alegre	Saccani et al, 2007	Cross-sectional	Brazil	Neuropsychomotor developmental delay	
8-	Risk factors for suspected neuropsychomotor developmental delay at 12 months of age	Halpern et al, 2000	Cross-sectional	Brazil	Neuropsychomotor developmental delay	
9-	Natural history of suspected developmental delay between 12 and 24 months of age in the 2004 Pelotas birth cohort	Moura et al, 2010	Longitudinal	Brazil	Global developmental delay	
10-	Epidemiology in child neurology: a study of the most common diagnoses	Lefévre et al, 1982	Descriptive	Brazil	Neuropsychomotor developmental delay	To diagnose children with developmental delay and avoid labeling them
11-	Diagnosis of developmental delay: the geneticists approach	Lunt, 1994	Theoretical	England	Developmental delay	
12-	Evaluation and management of children with low school achievement and attention deficit disorder	Araújo, 2002	Review	Brazil	Neuropsychomotor developmental delay	
13-	Global developmental delay and its relationship to cognitive	Grether, 2007	Theoretical	United States	Global developmental delay	To diagnose children with developmental delay and avoid labeling them
14-	Acquisition of functional skills in the mobility area in children attending an early stimulation program	Hallal et al, 2008	Cross-sectional	Brazil	Neuropsychomotor developmental delay	
15-	The infant or young child with developmental delay	First et al, 1994	Review	United States	Developmental delay	In children who do not have the motor milestones expected for their chronological age
16-	Classification of developmental delays	Petersen et al, 1998	Theoretical	United States	Developmental delay; psychomotor retardation	To identify children who have as the chief complaint the delay in meeting the developmental milestones in one or more areas of development
17-	Diagnostic evaluation of developmental delay/mental retardation: an overview	Bataglia et al, 2003	Review	United States	Developmental delay	Children younger than 5 years of age with suspected mental retardation
18-	Clinical genetic evaluation of the child with mental retardation or developmental delays	Moeschler et al, 2006	Theoretical	Canada	Developmental delay	
19-	“Is My Child Normal?”: Not all developmental problems are obvious. How to trust your instincts and tell if your child needs help	Costello et al, 2003	Theoretical	United States	Developmental delay	Children that show a peculiar variation of motor milestones when compared to the average child

**Table 4:** Articles organized according to the definition used for the term neuropsychomotor developmental delay.

Nº	Title	Author, year	Type of article	Country	Term	Definition
Nº	Title	Author, year	Type of article	Country	Term	Definition
1-	Child developmental delay and socio-economic disadvantage in Australia: a longitudinal study	Najman et al, 1992	Longitudinal	Australia	Developmental delay	Delay in language development, cognition, motor skills, and social skills within a particular culture
2-	Electrocochleography in children: study of 2,336 cases	Ramos et al, 1992	Cross-sectional	Brazil	Neuropsychomotor developmental delay	A combination of microcephaly; phonal abnormalities with atypical brain development
3-	Pediatric assessment of the child with developmental delay	Levy et al, 1993	Cross-sectional	United States	Developmental delay	Delay in two or more areas of child development with a standard deviation below the mean on developmental tests
4-	Diagnostic yield of the neurologic assessment of the developmentally delayed child	Majnemer et al, 1995	Longitudinal	Canada	Global developmental delay	
5-	The evaluation of the child with a global developmental delay	Shevell, 1998	Theoretical	Canada	Global developmental delay	
6-	Etiologic yield of subspecialists’ evaluation of young children with global developmental delay	Shevell, 2000	Cross-sectional	Canada	Global developmental delay	
7-	Practice parameter: evaluation of the child with global developmental delay	Shevell et al, 2003	Review	Canada	Global developmental delay	
8-	Developmental and functional outcomes in children with global developmental delay or developmental language impairment	Shevell et al, 2005	Longitudinal	Canada	Global developmental delay	
9-	Office evaluation of the child with developmental delay	Shevell, 2006	Theoretical	Canada	Global developmental delay	
10-	Analysis of clinical features predicting etiologic yield in the assessment of global developmental delay	Srour et al, 2006	Longitudinal	Canada	Global developmental delay	
11-	Investigation of global developmental delay	Mc Donald et al, 2006	Review	England	Global developmental delay	
12-	Global developmental delay and its relationship to cognitive skills	Grether, 2007	Theoretical	United States	Global developmental delay	
13-	Global developmental delay and its relationship to cognitive skills.	Riou et al, 2008	Longitudinal	Canada	Global developmental delay	Delay in two or more areas of child development with a standard deviation below the mean on developmental tests
14-	Investigation of developmental delay	Newton et al, 1995	Theoretical	England	Developmental delay	The child reaches the developmental milestones, but significantly more slowly than the average for other children, and may have future learning difficulties
15-	A preliminary study of creative music therapy in the treatment of children with developmental delay	Aldridge et al, 1995	Experimental	Germany	Developmental delay	A consequence of several physical, mental, and social difficulties
16-	Early intervention for young children with developmental delay: the Portage approach	Cameron, 1997	Review	United States	Global developmental delay	The result of several biological and environmental risk factors
17-	Study of primitive reflexes in normal preterm newborns in the first year of life	Olhweiler et al, 2005	Cross-sectional	Brazil	Neuropsychomotor developmental delay	
18-	Choice of medical investigations for developmental delay: a questionnaire survey	Gringras, 1998	Cross-sectional	United States	Developmental delay	A heterogeneous group of conditions resulting from the consequences of genetic, infectious, and chromosomal processes, and several other processes
19-	Genetics and developmental delay	Mac Millan, 1998	Theoretical	United States	Developmental delay	
20-	Motor profile in school children with learning difficulties	Rosa-Neto et al, 2005	Cross-sectional	Brazil	Neuropsychomotor developmental delay	A symptom that something is not as expected
21-	Early intervention in developmental delay	Kaur et al, 2006	Cross-sectional	India	Developmental delay	The physical, cognitive, language, social, or emotional delay, or a condition prone to delay of development
22-	Determinants of developmental delay in infants aged 12 months	Slykerman et al, 2007	Longitudinal	New Zealand	Developmental delay	The delay of motor milestones that may not necessarily be related to cognitive impairment, but rather to hypotonia or poor motor coordination, albeit without neurological signs, which does not justify a diagnosis of cerebral palsy
23-	Assessment of the neuropsychomotor development of children living in the outskirts of Porto Alegre	Saccani et al, 2007	Cross-sectional	Brazil	Neuropsychomotor developmental delay	A childhood development syndrome
24-	Characterization of motor performance in schoolchildren with attention deficit disorder and hyperactivity disorder	Toniolo et al, 2009	Cross-sectional	Brazil	Neuropsychomotor developmental delay	
25-	Developmental delay syndromes: psychometric testing before and after chiropractic treatment of 157 children	Cuthbert et al, 2009	Cross-sectional	United States	Developmental delay	
26-	Global developmental delay and mental retardation or intellectual disability: conceptualization, evaluation, and etiology	Shevell, 2008	Theoretical	Canada	Global developmental delay	A disorder or a dysfunction of childhood development
27-	Difficulties and facilities experienced by families in the care of children with cerebral palsy	Dantas et al, 2012	Descriptive	Brazil	Neuropsychomotor developmental delay	
28-	Global developmental delay – globally helpful?	Willians, 2010	Theoretical	United States	Global developmental delay	Delay in two or more fields of development considered significant when a discrepancy of 25% or more of the expected rate or a difference 1.5 to 2 standard deviations from the norm occurs in one or more areas of development in norm-referenced tests
29-	Present conceptualization of early childhood neurodevelopmental disabilities	Shevell, 2010	Theoretical	Canada	Global developmental delay	
30-	Developmental delay: timely identification and assessment	Pool et al, 2010	Review	United States	Developmental delay	
31-	Evaluation of children with global developmental delay: A prospective study at Sultan Qaboos University Hospital, Oman	Kou et al, 2012	Cross-sectional	Saudi Arabia	Global developmental delay	
32-	Characterization of the diagnostic profile and flow of a speech-language pathology service in child language within a public hospital	Mandrá et al, 2011	Cross-sectional	Brazil	Neuropsychomotor developmental delay	A comorbidity of child development
33-	Profile of special needs patients at a pediatric dentistry clinic	Menezes et al, 2011	Cross-sectional	Brazil	Neuropsychomotor developmental delay	A type of special needs in child development
34-	Monitoring of child development carried out in Brazil	Zeppone et al, 2012	Review	Brazil	Neuropsychomotor developmental delay	Progressive non-acquisition of motor and psychocognitive skills that progresses in the cephalocaudal and proximal-distal direction

After the coding and analysis of the articles, a conceptual map was constructed, according to the specifications of Novak,^12^ the creator of this tool. The conceptual map is considered a knowledge-structuring tool. It can be understood as a visual representation used to share meanings, and relies heavily on the meaningful learning theory of David Ausubel,^13^ which proposes that human beings organize their knowledge through the hierarchization of concepts.

There are several types of maps, which can be used in different situations, according to the specific purpose. The conceptual map used in this review is the system type, which organizes the information in a format similar to a flow chart and shows the various associations between the concepts. The CmapTools software was used to create the conceptual map.^14^

## Results

Of the 71 articles selected for the review, 55 (77.5%) were international and 16 were national publications (22.5%). The terms global developmental delay and developmental delay were the most frequent in the international literature and emerged in the mid-1940s and 1960s, respectively, gaining strength in the 1990s. In Brazil, the terms *retardo do DNPM* and *atraso do DNPM* started to be used in the scientific area in the 1980s, but it was in the last decade that they started to be frequently mentioned in the literature.

### 1- Regarding the population to which the term was applied (Table 2)

As mentioned in 23 articles, 13 types of clinical conditions showed developmental delay as a characteristic. In the international literature, the first time the term was mentioned was to refer to preterm children and those with mental retardation. Subsequently, other conditions that included developmental delay were encompassed, such as cerebral palsy, autism, chromosomal abnormalities, and congenital abnormalities. In the late 1990s, it was expanded to the population of children who had no defined underlying pathology, but who had some kind of developmental delay as a characteristic. 

In Brazil, the first time the term was mentioned, it also referred to children with mental retardation and, subsequently, to children with sensory impairments.

### Regarding use of the term (Table 3)

A total of 19 articles were found, which showed seven types of situations to which the term was applied. Internationally, it started to be used for children with neurological disorders that had atypical development or for children who did not reach the motor development milestones expected for their chronological age. Subsequently, the concept of the delay was operationalized by means of norm-referenced tests for development. At the end of the 1990s, the term started to be used in children younger than five years, pending definitive diagnosis, as well as with diagnosis, albeit with no specific criteria.

In Brazil, the term started to be used in the 1980s as a diagnosis for children with mental retardation and, from the 1990s on, for any child that showed some type of developmental delay. Only more recently the term has been used for children with low scores on norm-referenced tests.

### Regarding definition of the term (Table 4)

Thirty-four articles included definitions, in which 16 different concepts were identified. In the international literature, the term definitions started to appear in the 1990s, and the concept initially referred to a slower development than other children from the same culture; such delayed development was attributed to a heterogeneous group of biological and environmental factors.

Since 2000, other term definitions emerged based on the motor milestones of development, which could be justified by hypotonia or poor motor coordination without specific cause, and the delay should be quantified by applying the norm-referenced tests. Recently, the term was conceptualized by the American Academy of Neurology and the Child Neurology Committee^11^ as a delay in two or more areas of development, considered significant when a discrepancy of 25% or more of the expected rate occurs, or a difference of 1.5-2.0 standard deviations from the norm in one or more areas of development in norm-referenced tests.

In Brazil, in the early 1990s, the term was conceptualized as a combination of microcephaly, with phonal abnormalities and atypical brain development. Over the last decade, it received several meanings, as a symptom, syndrome, disorder, comorbidity, and even as a special need. The most recent Brazilian article defines the term as the progressive non-acquisition of motor and psychocognitive skills in an orderly and sequential manner, which progresses in the cephalocaudal direction, and from the proximal to the distal.

**Figure 2:**
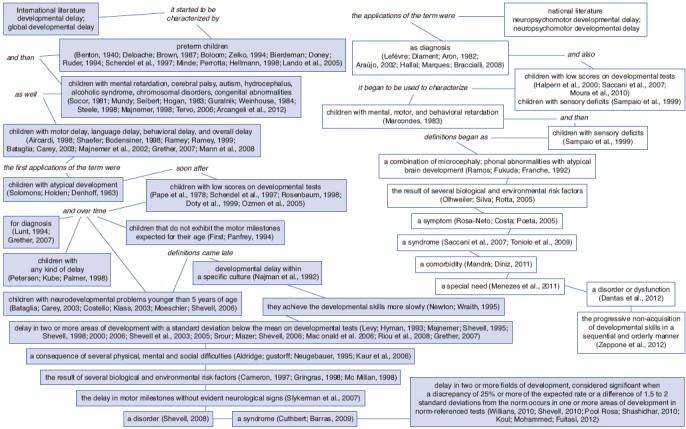
Conceptual map of the term neuropsychomotor development delay.

Based on the analysis of tables and information obtained from the articles, the conceptual map was constructed (Fig. 2), showing the origin of the terms related to developmental delay and evolution over time.

## Discussion

The present review shows that, in the international literature, the terms related to the NPMD delay started to be used in studies on cognitive development of preterm children. The oldest study identified, by Benton^15^ in 1940, used the Stanford-Binet intelligence test to characterize developmental delay in children born prematurely. According to the author, although preterm children were not intellectually inferior, they suffered from anxiety, nervousness, and fatigue, resulting in distraction and poor concentration. Thus, it can be observed that, in the scientific literature, the early works on developmental delay used cognitive tests,^16^ soon followed by the extensive work of Gesell,^17^ who created the first scale of developmental milestones by age range in 1940 and gave rise to further studies that sought to characterize developmental delay in several populations.

Gesell's studies were disseminated worldwide, inspiring the creation of several currently used development tests and influencing the use of the term developmental delay, thus one of the most frequent use scenarios found in this study was the operationalization of the term through developmental tests.^18^^-^ 22 For instance, in one of the first references located, in 1978 Pape and colleagues^19^ adopted as criteria for developmental delay in infants the development index below that expected for age assessed according to first version of the Bayley Scales of Infant Development,^23^ a test based on the work of Gesell.

Nevertheless, although the term developmental delay has emerged from the neuromaturational perspective^17^ and has been widely used in the area of child health, there is no term consensus regarding both the population to which it is applied (Table 2)^15,24^ and the term use (Table 3).^25^^,^ 26 In both situations, the term is mentioned in a generalized and excessively encompassing manner; this range of possibilities can be justified by the most widely used method to identify children with delay: developmental screening.^18^^-^ 22

In fact, developmental screening is the best option to screen children with developmental problems, as it is a fast procedure suitable for application to large populations of children of several age groups.^27^ However, some reviewed studies^5^^,^ 28 ^,^ 29 mention that *ad hoc* assessments of development in children younger than five years of age are unreliable to establish a definitive diagnosis, suggesting that the term developmental delay should be used as a temporary diagnosis. 

One problem is that this use, even when it is temporary, gives the impression of a relatively benign condition that is resolved over time. However, the studies reviewed^30^^-^ 32 on the outcome of children that had developmental delay in the first years of life show persistent developmental difficulties. Newton and Wraith^33^ stated that most children younger than 5 years of age with developmental delay will have some type of learning disability at school age, and thus, it is important to attain a correct diagnosis.

Some authors^29^^,^ 34 suggest that, considering these remaining problems, development monitoring in this population would be beneficial. Such an approach would involve periodic reassessments at key points of development, with the aim not only to identify problems as they arise, with referral to early intervention, but also to assist the achievement of a definitive diagnosis.^27^^,^ 35

Another aspect identified in this review refers to the term definitions (Table 4), which only started to emerge in the mid-1990s, but in a quite heterogeneous manner. This need to better define the concept may have been caused by a lack of standardization, which became unsustainable with the significant increase in publications from this period onward.

Some authors started to conceptualize the term based on the results of their studies, such as Najman et al^36^ in 1992, when they studied the development of Australian children from the perspective of socioeconomic inequality, considered a national problem in the country, and found a higher prevalence of developmental delay in children who had mothers with low educational and socioeconomic levels. Based on this finding, the authors conceptualized the delay as the result of biological and environmental factors within a specific culture. That is, different factors interacting with the child's development, influencing the acquisition of motor, cognitive, language, and social skills.

In the last decade, international publications have shown more standardized definitions, consistent with the scientific advances in the area. The most recent concept that was suggested by the medical community^10^^,^ 37 ^,^ 38 was operationally defined by the American Academy of Neurology and the Child Neurology Committee.^10^ The definition, as mentioned before, encourages the use of validated tests, with norms and reference criteria to support the reliable measurement of relevant clinical data that can confirm the developmental delay.^10^

The Committee also suggests tests that can be used for each domain - motor domain: Alberta Infant Motor Scale; Peabody Developmental Motor Scale; Bruininks-Oseretsky Test of Motor Proficiency; phonal/language domain: Language-Peabody Picture Vocabulary Test; Expressive One Word Vocabulary Test; Clinical Linguistic Auditory Milestone Scale; Clinical Evaluation of Language Fundamentals; behavioral domain: Vineland Adaptive Behavior Scales, Pediatric Evaluation of Disability Inventory, Wee Functional Independence Measure; and for multiple domains: Batelle Developmental Inventory.^10^

As for the definitions in Brazil, over the decades little conceptual evolution can be observed, but many meanings.^39^^-^ 45 The implicit concept in the term also follows the neuromaturational perspective;^17^ however, it does not have quantitative parameters as found in other countries.^10^ That is, the Brazilian definitions do not encourage the use of tools for development assessment, which is justified by the small number of child development tests that have been validated and standardized for the Brazilian population. 

In fact, in Brazil, the use of the term in scientific publications began more recently,^46^ in 1982, than in the international literature^15^ and very specific names, uses, and definitions were found in the literature. Starting with the terminology, in Brazil the first mention found was NPMD retardation,^47^ with the addition of the word psychomotor, which is not used in the international literature. This term originated in the mid-1950s, used by the neurologist Lefévre^48^ in his habilitation thesis (1950), in which he argued that, for the child to develop neuropsychomotor skills, he/she needs both neural growth and maturation, and psychological and motor aspects. In his thesis, Lefévre presents the first scale for neuromotor assessment for Brazilian children, based on the works of Ozeretski (1936)^49^ and the psychiatrist Ajuriaguerra (1948),^50^ where, possibly, the term psychomotor came from.

The closest term to NPMD retardation, found in the reviewed works written in the English language, was psychomotor retardation, used by Fenichel (quoting Petersen, Kube, Palmer, 1998),^5^ to refer to "children with mild mental retardation and motor delay, caused by mild hypotonia or poor motor coordination rather than low cognitive function." It is noteworthy that the term psychomotor is used only in Brazil. As the work of Lefevre was very influential, it possibly determined the future use of the term.

In the first Brazilian scientific publications, the term NPMD delay was used as a diagnostic term to refer children to with cognitive impairment and mild motor delay, widely used by neurologists in Brazil. For instance, in 1982, Lefèvre and Diament,^46^ in a study performed with the objective of mapping the most common diagnoses in child neurology in Brazil, found that among the 16 most frequent diagnoses, NPMD delay was the third most common. Shortly thereafter, this term became known as delayed NPMD, described by Marcondes,^51^ but keeping the same emphasis on the use, as a way to soften the terminology, since the term retardation was associated with children with severe impairment. 

The conceptual map (Fig. 2) shows that there are several definitions, and that the international literature, in addition to showing a richer repertoire, is more concerned with the standardization of the term definition and the incentive to research the cause of the delay, investing, in more recent studies, in specific diagnostic tests.^52^ This is not observed in Brazil, as in addition to the fact that the literature on the topic is recent, it is also scarce, with very specific definitions and studies more focused on the risk factors for the delay.

In short, it is observed that the developmental delay is discussed in the international and national literature under different names, and has different applications and heterogeneous concepts. However, studies call attention to a fact in common, that *something is not going well* with the child, as he/she does not follow the expected sequence of important acquisitions for development. Internationally, an investment in definition standardization has been observed, which was not seen in national publications.^10^^,^ 11 In other countries, the recommendation is to use the term in children younger than five years of age with developmental abnormalities, always identified by standardized tests,^5^^,^ 28 ^,^ 29 and to employ periodic reviews with the aid of additional tests during the first years of life, in an attempt to find the cause of the delay and establish the final diagnosis.^29^^,^ 53

## Conclusion

A more precise definition of NPMD delay is essential for adequate care provision. However, the use of this term has generated difficulties in direct clinical decisions on the levels of assessment, intervention, and definition of prognosis of small children. Internationally, methods to improve communication between professionals, with standardized definition of the term and its use in very specific situations, have been proposed.

In Brazil, it is necessary to invest in both the standardization of the term, as well as in well-documented follow-up programs of children with suspected delay. The monitoring of development is a process that can help professionals and parents to understand what happens to the child, up to the establishment of the final diagnosis, as the term NPMD delay is more appropriately used as a temporary diagnosis.
