# Supplementary material for: Neuropsychomotor developmental delay: conceptual map, term definitions, uses and limitations
Source: Rev Paul Pediatr. 2015 Mar;33(1):88–103. doi: 10.1016/j.rpped.2014.04.009 (PMC4436961; doi:10.1016/j.rpped.2014.04.009)
Supplement: Translation

# Translation: Atraso do desenvolvimento neuropsicomotor: mapa conceitual, definições, usos e limitações do termo

**Authors:** Lílian de Fátima Dornelas, Neuza Maria de Castro Duarte, Lívia de Castro Magalhães

**Affiliations:** a Universidade Federal de Minas Gerais (UFMG), Belo Horizonte, MG, Brasil; b Associação de Assistência à Criança Deficiente de Minas Gerais (AACD-MG), Uberlândia, MG, Brasil

**Keywords:** Desenvolvimento infantil, Transtornos globais do desenvolvimento infantil, Revisão da literatura

## Abstract

**OBJETIVO:**

Resgatar a origem do termo *atraso do desenvolvimento neuropsicomotor (DNPM)*, sua evolução conceitual ao longo do tempo e construir mapa conceitual do termo com base em busca bibliográfica.

**FONTES DE DADOS:**

Foi realizada busca nas bases de dados eletrônicas do Portal da Capes, que incluem Scielo Brazil, Web of Science, Science Direct, OneFile (GALE), Pubmed (Medline), Whiley Online e Springer, referente a Janeiro/1940-Janeiro/2013. Palavras-chave: *atraso e retardo do DNPM*, *developmental delay* e *global developmental delay*. Foram selecionados 71 artigos e construído o mapa conceitual do termo.

**SÍNTESE DE DADOS:**

Das 71 referências, 55 eram internacionais e 16 nacionais. Os termos mais encontrados foram *global developmental delay e developmental delay* na literatura internacional e *retardo* e *atraso do DNPM* no Brasil. Internacionalmente, o termo surgiu em meados da década de 40 ganhando força nos anos 90. No Brasil, o termo começou a ser usado na década de 80 e vem sendo frequentemente citado na literatura. O atraso é citado em 23 trabalhos como característica presente em 13 tipos de condições clínicas. Com relação ao uso, foram encontrados 19 estudos, com sete situações de uso. Dentre os artigos revisados, 34 deles apresentaram definições, sendo identificados 16 conceitos diferentes.

**CONCLUSÕES:**

O atraso do desenvolvimento é abordado na literatura internacional e nacional sob diversos nomes, diferentes aplicações e conceitos heterogêneos. Internacionalmente, apontam-se caminhos para melhorar a comunicação entre profissionais, com definição padronizada do termo e uso em situações específicas até o quinto ano de vida, o que não foi encontrado nas publicações nacionais.

## Introdução

*Artur nasceu com idade gestacional de 32 semanas pesando 2.100 gramas. Com 6 meses de vida foi encaminhado pelo pediatra para a fisioterapia devido a atraso do desenvolvimento neuropsicomotor (DNPM), pois, segundo a mãe,"ele não firmava o pescoço e era molinho". A mãe foi informada que não precisava se preocupar, pois não era nada grave e, de fato, Artur apresentou rápida evolução motora, recebendo alta dos atendimentos. Atualmente, Artur está com 7 anos de idade e tem dificuldade para usar talheres, amarrar sapatos e não realiza sozinho tarefas do toalete. Ele não consegue jogar bola, mas adora videogames. Segundo sua mãe, Artur é uma criança tranquila, que anda, fala, enxerga, ouve e entende o que se fala com ele normalmente, mas tem pouca iniciativa, é muito dependente e tem dificuldade em adaptar-se às pessoas e ambientes novos. Na escola, segundo a professora, é uma criança tímida, mas que interage com os colegas e participa de todas as atividades necessitando de assistência mínima. Ele está aprendendo a ler e a escrever, mas é mais lento que os colegas e desatento. Os pais estão confusos, pois a criança persiste com o diagnóstico de atraso do DNPM, que não o qualifica para receber suporte especializado*.

Estima-se que, em todo o mundo, 200 milhões de crianças menores de cinco anos de idade estão sob risco de não atingir seu pleno desenvolvimento.^1^ A prevalência do atraso do desenvolvimento é, em grande parte, uma incógnita, mas dados da Organização Mundial de Saúde (OMS) indicam que 10% da população de qualquer país é constituída por pessoas com algum tipo de deficiência, com uma taxa de 4,5% entre aquelas com até cinco anos de idade.^1^ No Brasil,^2^ foi detectada diminuição da prevalência de crianças com atraso do desenvolvimento, o que se justifica pelos avanços nos cuidados neonatais, pela ampliação da cobertura de assistência à criança no primeiro ano de vida, ocorrido nas últimas décadas nos hospitais dos grandes centros e do interior do país, além da elevação das condições socioeconômicas da população. Porém, esses mesmos fatores provocaram uma situação paradoxal, pois a maior sobrevivência de bebês de risco, especialmente os prematuros, está associada a aumento da morbidade, como as sequelas no neurodesenvolvimento, gerando nova demanda para o pediatra e demais profissionais da saúde.

O atraso do desenvolvimento está associado a várias condições da infância, desde a concepção, gravidez e parto, decorrentes de fatores adversos como a subnutrição, agravos neurológicos, como a encefalopatia crônica da infância (paralisia cerebral), e genéticos, como a síndrome de Down. O atraso pode ser também uma condição transitória, não sendo possível definir qual será o desfecho do desenvolvimento da criança, o que pressupõe o acompanhamento com avaliações periódicas. Observa-se, ainda, não ser incomum encontrar o termo como diagnóstico, como no caso de Artur, sem uma definição mais objetiva do que está acontecendo com a criança.

Embora o termo atraso do desenvolvimento seja bastante utilizado na área da saúde da criança, sendo muito empregado clinicamente e citado na literatura, é interessante notar, como discutido por Aircadi,^3^ em 1998, que esse termo não consta como título de capítulo e nos índices da maioria dos livros de neurologia infantil, nem aparece na Classificação Internacional de Doenças - 10^a^ revisão (CID-10) e no Manual de Diagnóstico e Estatística das Perturbações Mentais - 4^a^ versão (DSM-IV).

Mas o que significa ter atraso do desenvolvimento? Pelo *Dictionary of Developmental Disabilities Terminology*, 4 atraso do desenvolvimento é uma condição em que a criança não está se desenvolvendo e/ou não alcança habilidades de acordo com a sequência de estágios pré-determinados. Porém, esta definição não é consensual e a falta de padronização do conceito tem gerado discordâncias entre os profissionais da área, levando a situações muito variadas de uso e uma infinidade de termos (ex.: atraso do desenvolvimento, atraso do desenvolvimento neuropsicomotor, retardo mental, retardo do desenvolvimento neuropsicomotor, atraso do desenvolvimento global), que parecem não apresentar o mesmo significado, embora muitas vezes sejam usados de maneira semelhante. 3 ^,^ 5 ^and^ 6

Na realidade, é um termo que tem confundido os profissionais e principalmente os pais, pois o termo atraso passa a percepção de "demora", que "vai se chegar a algo" ou, ainda, que o desenvolvimento é lento, mas que a criança irá alcançar seu destino final, ou seja, que o problema é temporário e o prognóstico é favorável.^7^^,^ 8 ^and^ 9 O que nem sempre ocorre, como no caso de Artur, sendo o termo utilizado ao longo dos anos de maneira genérica, não atuando como instrumento de comunicação, trazendo insatisfação para os pais, por não saberem o que seu filho tem, e frustração na escola, pois sem um diagnóstico específico, a criança não é elegível para receber suporte pedagógico especializado ou assistência pela equipe de saúde.

Assim, o termo mais parece ser subproduto de dificuldades conceituais e metodológicas em definir e medir de forma confiável as habilidades de crianças jovens, pois pode ser aplicado de maneira indiferenciada tanto a uma criança com atraso leve como com grave comprometimento. Um bebê, por exemplo, que apresentar atraso na motricidade fina e na linguagem poderá receber o mesmo rótulo que um bebê com grave atraso motor e cognitivo, ou seja, serão tratados como apresentando uma entidade homogênea, tanto em termos de causa quanto de prognóstico.^7^ ^and^ 8 Na prática, o médico nem sempre conta com instrumentos adequados, o que inclui testes válidos e confiáveis de desenvolvimento, ou com o suporte de equipe interdisciplinar que colabore para o diagnóstico. Além disso, medir o atraso do desenvolvimento exige habilidade de reconhecer que as trajetórias do desenvolvimento são invariavelmente individualizadas, havendo variações dentro do que pode ser aceito como "normal" e "não normal",^10^ ^and^ 11 o que implica na necessidade de contato mais prolongado para se conhecer o contexto de vida da criança.

Dado o uso frequente e as confusões conceituais relacionadas ao uso do termo atraso no desenvolvimento, o objetivo deste estudo foi buscar informações, por meio de busca bibliográfica, sobre o termo *atraso do DNPM*, visando resgatar sua origem e evolução conceitual ao longo do tempo, como documentado em artigos científicos. Como forma de organizar o conhecimento, foi construído mapa conceitual para dar uma visão da complexidade do uso dessa terminologia.

## Método

Inicialmente foi realizada uma busca pelo tema no Portal da Capes com uso do termo *atraso do desenvolvimento* (*developmental delay*) com o objetivo de identificar as bases de dados que indexam artigos sobre o tema proposto. As bases de dados mais frequentes foram: *Scielo Brazil, Web of Science, Science Direct, OneFile (GALE), Pubmed (Medline), Whiley Online e Springer*. Em seguida, foram realizadas buscas com termos específicos em cada base conforme descrito na tabela 1. As buscas e codificação dos dados foram feitas pela primeira autora.

**Tabela 1:** Base de dados eletrônicas com os termos utilizados e quantidade de documentos encontrados

Base de dados	Termos utilizados	Número de documentos encontrados
Scielo Brazil	atraso do desenvolvimento; atraso do desenvolvimento neuropsicomotor; retardo do desenvolvimento neuropsicomotor, atraso do desenvolvimento global; retardo mental	498
Web of Science	developmental delay* AND child; global developmental delay*; neuropsychomotor developmental delay*; neuropsychomotor developmental retardation*; mental retardation* AND child; neurodevelopmental disabilities* AND child; developmental disorder* AND child *	7.545
Science Direct	developmental delay AND child; global developmental delay; neuropsychomotor developmental delay; neuropsychomotor developmental retardation; mental retardation AND child; neurodevelopmental disabilities AND child; developmental disorder AND child	3.672
Pubmed (Medline)	developmental delay child; global developmental delay; neuropsychomotor developmental delay; neuropsychomotor developmental retardation; mental retardation child; neurodevelopmental disabilities child; developmental disorder child	8.974
Whiley Online Library	developmental delay AND child; global developmental delay AND child; neuropsychomotor developmental delay; neuropsychomotor developmental retardation; mental retardation AND child; neurodevelopmental disabilities AND child; developmental disorder AND child	3.662
Springer	developmental delay; global developmental delay; neuropsychomotor developmental delay; neuropsychomotor developmental retardation; mental retardation; neurodevelopmental disabilities; developmental disorder	4.505
OneFile (GALE)	children with developmental delays; global developmental delay; neuropsychomotor developmental delay; neuropsychomotor developmental retardation; mental retardation AND children's; neurodevelopmental disabilities; developmental disorders	675
Total		29.531

Com o objetivo de resgatar a origem do termo, a estratégia de busca não teve limite de data, incluindo desde os primeiros registros sobre o tema, publicados em 1940, até janeiro de 2013, resultando em 29.531 documentos. Visando focar em termos mais específicos, em cada base foi utilizado o recurso *filtrar meus resultados*, por tópicos. Por meio de filtro, foi observado que, das palavras-chave utilizadas, os termos *developmental delay* e *global developmental delay* para a literatura internacional e *atraso do desenvolvimento*, *atraso do DNPM* e *retardo do DNPM* nos bancos de dados nacionais foram os mais adequados para englobar e encontrar o maior número de artigos relacionados ao objetivo proposto. Assim, após utilização do filtro para estes termos, foram selecionados 3.679 estudos. Posteriormente, os títulos e os resumos dos trabalhos localizados foram analisados, para eliminar aqueles não relacionados ao tema proposto. Para selecionar os estudos para a leitura na íntegra, como ilustrado na figura 1, foram aplicados os seguintes critérios de inclusão e exclusão até se chegar aos trabalhos finais:

**Figura 1:** Etapas da busca bibliográfica.

## Critérios de inclusão

- Artigos publicados em inglês e português; artigos originais, de revisão e os especiais (teóricos);
- Artigos que utilizaram os termos *developmental delay* e *global developmental delay* para a literatura internacional e *atraso do DNPM e retardo do DNPM* na literatura nacional, que incluíam pelo menos uma das três informações abaixo:
- População - descrição ou citação do tipo de transtorno ou população enquadrada no termo;
- Uso - descrição da situação ou critério utilizado para empregar o termo;
- Definição - apresentação de definição ou conceito, explicando o significado do termo.

**Critérios de exclusão**: tópicos que não estavam relacionados com as palavras-chave utilizadas, estudos que apenas citaram o termo sem qualquer informação, relato de caso, resenhas, cartas ao editor, depoimentos, entrevistas, ponto de vista, editoriais, atas de congresso e comentários de jornais. Livros e capítulos de livros foram excluídos devido à dificuldade de se localizar tanto os registros como o material na íntegra, eletronicamente, especialmente os mais antigos.

Os artigos selecionados foram lidos na íntegra para extração detalhada dos dados, que foram organizados em três tabela, de acordo com o tipo de informação obtida: a primeiro aborda a população enquadrada no termo (tabela 2), a segunda descreve como o termo foi usado (tabela 3), e a última, as definições do termo (tabela 4). Em cada tabela foi incluída informação sobre o título, autor e ano, tipo de artigo, país, termo utilizado e a informação específica sobre o termo. Com o intuito de abarcar o maior número de informações sobre o uso do termo considerando uma perspectiva histórica, optou-se por não avaliar a qualidade metodológica dos artigos, porém, todos os estudos se enquadraram nos critérios de inclusão e exclusão estabelecidos para a presente revisão. As listas de referências bibliográficas dos artigos foram examinadas, na tentativa de localizar fontes de informação e referencial teórico que deram origem as definições e usos do termo utilizados nos artigos.

**Tabela 2:** Artigos organizados segundo a população enquadrada no termo atraso do DNPM

N°	Título	Autor, ano	Desenho	País	Termo	População
1	Mental development of prematurely born children.	Benton, 1940	Transversal	Estados Unidos	Global developmental delay	Criança pretermo.
2	Impact of delayed development in premature infants on mother-infant interaction: A prospective investigation.	Minde et al, 1998	Longitudinal	Canadá	Developmental delay	
3	Relation between very low birth weight and developmental delay among preschool children without disabilities.	Schendel et al, 1997	Transversal	Estados Unidos	Developmental delay	
4	Developmental delay at 12 months in children born extremely preterm.	Lando et al, 2005	Longitudinal	Estados Unidos	Developmental delay	
5	In pursuit of potential: a discussion of developmental delay, structuralization and one child's effotrs at mastery.	Socor, 1981	Teórico	Estados Unidos	Developmental delay	Criança com paralisia cerebral.
6	Novelty responding and behavioral development in young, developmentally delayed children.	Mundy et al, 1983	Transversal	Estados Unidos	Developmental delay	Criança com alterações cromossômicas e congênitas, com hidrocefalia, com autismo,com síndrome alcóolica e criança pretermo
7	Preschool Children with developmental delays:nursing intervention.	Steele, 1998	Descritivo	Estados Unidos	Developmental delay	
8	Benefits of early intervention for children with developmental disabilities.	Majnemer, 1998	Teórico	Canadá	Global developmental delay	
9	Neurodevelopmental delay in small babies at term: a systematic review.	Arcangeli et al, 2012	Revisão	Reino Unido	Developmental delay	
10	Semiologia do crescimento deficiente: roteiro de diagnóstico.	Marcondes, 1983	Teórico	Brasil	Atraso do desenvolvimento neuropsicomotor	Criança com retardo mental.
11	The etiology of developmental delay.	Aircardi, 1998	Teórico	Inglaterra	Developmental delay	
12	Radiological findings in developmental delay.	Shaefer et al, 1998	Teórico	Estados Unidos	Developmental delay	
13	Early experience and early intervention for children at risk for developmental delay and mental reatardation.	Ramey et al, 1999	Revisão	Estados Unidos	Developmental delay	
14	Diagnostic evaluation of developmental delay/mental retardation: an overview.	Bataglia et al, 2003	Revisão	Estados Unidos	Developmental delay	
15	Peer-related social interactions of developmentally delayed young children: development and characteristics.	Guranilk et al, 1984	Longitudinal	Estados Unidos	Developmental delay	Criança com alterações cromossômicas e congênitas e criança pretermo.
16	Identifying patterns of developmental delays can help diagnose neurodevelopmental disorders.	Tervo, 2006	Teórico	Reino Unido	Global developmental delay	
17	Differences in the memory-based searching of delayed and normally developing young children	Deloache et al, 1987	Transversal	Estados Unidos	Developmental delay	Criança com paralisia cerebral, com atraso da linguagem e criança pretermo.
18	Variability in adaptive behavior in children with developmental delay.	Bloom et al, 1994	Transversal	Estados Unidos	Developmental delay	Criança com retardo mental e criança pretermo.
19	The negative effects of positive reinforcement in teaching children with developmental delay.	Bierdeman et al, 1994	Transversal	Canadá	Developmental delay	
20	Avaliaçäo do retardo do neurodesenvolvimento em crianças especiais em serviço universitário de visäo subnormal.	Sampaio et al, 1999	Transversal	Brasil	Retardo do desenvolimento neuropsicomotor	Criança com deficiência sensorial.
21	Early rehabilitation service utilization patterns in young children with developmental delays.	Majnemer et al, 2002	Longitudinal	Canadá	Global developmental delay	Criança com atraso global, com atraso motor, com atraso da linguagem e com autismo.
22	Global developmental delay and its relationship to cognitive.	Grether, 2007	Teórico	Estados Unidos	Global developmental delay	Criança com retardo mental, com atraso motor.
23	Does race influence age of diagnosis for children with developmental delay?	Mann et al, 2008	Longitudinal	Estados Unidos	Developmental delay	Criança com paralisia cerebral, com atraso da linguagem e com deficiência sensorial.

**Tabela 3:** Artigos organizados segundo a forma de uso do termo atraso do DNPM

N°	Título	Autor, ano	Desenho	País	Termo	Uso do termo
1	The changing picture of cerebral dysfunction in early childhood.	Solomons et al, 1963	Longitudinal	Estados Unidos	Developmental delay	Em crianças que não apresentam desenvolvimento típico sem sinais neurológicos evidentes que possam considerá-las com paralisia cerebral.
2	The status at two years of low-birth-weight infants born in 1974 with birth weights of less than 1,001 gm.	Pape et al, 1978	Longitudinal	Canadá	Developmental delay	Em crianças que apresentam baixa pontuação em testes de desenvolvimento.
3	Relation between very low birth weight and developmental delay among preschool children without disabilities.	Schendel et al, 1997	Transversal	Estados Unidos	Developmental delay	
4	Screening tests and standardized assessments used to identify and characterize developmental delays.	Rosenbaum, 1998	Teórico	Canadá	Developmental delay	
5	Effects of testing context on ball skill performance in 5 year old children with or without developmental delay	Doty et al, 1999	Transversal	Estados Unidos	Developmental delay	
6	Etiologic evaluation in 247 children with global developmental delay at Istanbul Turkey.	Ozmen et al, 2005	Transversal	Turquia	Global Developmental delay	
7	Avaliação do desenvolvimento neuropsicomotor em crianças de um bairro da periferia de Porto Alegre.	Saccani et al, 2007	Transversal	Brasil	Atraso do desenvolvimento neuropsicomotor	
8	Fatores de risco para suspeita de atraso no desenvolvimento neuropsicomotor aos 12 meses de vida.	Halpern et al, 2000	Transversal	Brasil	Atraso do desenvolvimento neuropsicomotor	
9	Natural history of suspected developmental delay between 12 and 24 months of age in the 2004 Pelotas birth cohort.	Moura et al, 2010	Longitudinal	Brasil	Atraso do desenvolvimento global	
10	Epidemiologia em neurologia infantil: estudo dos diagnósticos mais comuns.	Lefévre et al, 1982	Descritivo	Brasil	Retardo do desenvolvimento neuropsicomotor	Para diagnosticar crianças que apresentam retardo do desenvolvimento e evitar de rotulá-las.
11	Diagnosis of developmental delay: the geneticists approach.	Lunt, 1994	Teórico	Inglaterra	Developmental delay	
12	Avaliação e manejo da criança com dificuldade escolar e distúrbio de atenção.	Araújo, 2002	Revisão	Brasil	Atraso do desenvolvimento neuropsicomotor	
13	Global developmental delay and its relationship to cognitive.	Grether, 2007	Teórico	Estados Unidos	Global developmental delay	Para diagnosticar crianças que apresentam retardo do desenvolvimento e evitar de rotulá-las.
14	Aquisição de habilidades funcionais na área de mobilidade em crianças atendidas em um programa de estimulação precoce.	Hallal et al, 2008	Transversal	Brasil	Atraso do desenvolvimento neuropsicomotor	
15	The infant or young child with developmental delay.	First et al, 1994	Revisão	Estados Unidos	Developmental delay	Em crianças que não apresentam os marcos motores esperados para a sua idade cronológica.
16	Classification of Developmental Delays.	Petersen et al, 1998	Teórico	Estados Unidos	Developmental delay; psychomotor retardation	Para identificar criança que apresente como queixa principal o atraso no cumprimento dos marcos do desenvolvimento em umou mais domínios do desenvolvimento.
17	Diagnostic evaluation of developmental delay/mental retardation: an overview.	Bataglia et al, 2003	Revisão	Estados Unidos	Developmental delay	Em crianças abaixo de 5 anos de idade com suspeita de retardo mental.
18	Clinical genetic evaluation of the child with mental retardation or developmental delays.	Moeschler et al, 2006	Teórico	Canadá	Developmental delay	
19	Is My Child Normal?“: Not all developmental problems are obvious. How to trust your instincts and tell if your child needs help.	Costello et al, 2003	Teórico	Estados Unidos	Developmental delay	Em crianças que variam os marcos motores de maneira peculiar em relação a média das crianças.

**Tabela 4:** Artigos organizados segundo a definição usada para o termo atraso do DNPM

N°	Título	Autor, ano	Desenho	País	Termo	Definição
1	Child developmental delay and socio-economic disadvantage in Australia: a longidutinal study.	Najman et al, 1992	Longitudinal	Austrália	Developmental delay	É o atraso do desenvolvimento da linguagem, da cognição, da motricidade e das habilidades sociais dentro de uma cultura específica.
2	Eletrococleografia em crianças: estudo de 2.336 casos.	Ramos et al, 1992	Transversal	Brasil	Retardo do desenvolvimento neuropsicomotor	É uma combinação de microcefalia, anormalidades fonais com desenvolvimento cerebral atípico.
3	Pediatric assessment of the child with developmental delay.	Levy et al, 1993	Transversal	Estados Unidos	Developmental delay	É o atraso em dois ou mais áreas do desenvolvimento infantil com desvio-padrão abaixo da média nos testes de desenvolvimento.
4	Diagnostic yield of the neurologic assessment of the developmentally delayed child.	Majnemer et al, 1995	Longitudinal	Canadá	Global developmental delay	
5	The evaluation of the child with a global developmental delay.	Shevell, 1998	Teórico	Canadá	Global developmental delay	
6	Etiologic yield of subspecialists’ evaluation of young children with global developmental delay.	Shevell, 2000	Transversal	Canadá	Global developmental delay	
7	Practice parameter: evaluation of the child with global developmental delay.	Shevell et al, 2003	Revisão	Canadá	Global developmental delay	
8	Developmental and functional outcomes in children with global developmental delay or developmental language impairment.	Shevell et al, 2005	Longitudinal	Canadá	Global developmental delay	
9	Office evaluation of the child with developmental delay.	Shevell, 2006	Teórico	Canadá	Global developmental delay	
10	Analysis of Clinical Features Predicting Etiologic Yield in the Assessment of global developmental delay.	Srour et al, 2006	Longitudinal	Canadá	Global developmental delay	
11	Investigation of global developmental delay.	Mc Donald et al, 2006	Revisão	Inglaterra	Global developmental delay	
12	Global developmental delay and its relationship to cognitive skills	Grether, 2007	Teórico	Estados Unidos	Global developmental delay	
13	Global developmental delay and it is relationship to cognitive skills.	Riou et al, 2008	Longitudinal	Canadá	Global developmental delay	É o atraso em dois ou mais áreas do desenvolvimento infantil com desvio-padrão abaixo da média nos testes de desenvolvimento.
14	Investigation of developmental delay.	Newton et al, 1995	Teórico	Inglaterra	Developmental delay	É quando atinge as habilidades do desenvolvimento, mas de maneira significativamente mais lenta que a média das outras crianças e pode apresentar dificuldades futuras de aprendizagem.
15	A preliminary study of creative music therapy in the treatment of children with developmental delay.	Aldridge et al, 1995	Experimental	Alemanha	Developmental delay	É uma consequência de várias dificuldades físicas, mentais e sociais.
16	Early intervention for young children with developmental delay: the Portage approach	Cameron, 1997	Revisão	Estados Unidos	Global developmental delay	É o resultado de uma variedade de fatores de risco biológicos e ambientais.
17	Estudo dos reflexos primitivos em pacientes recém-nascidos pré-termo normais no primeiro ano de vida.	Olhweiler et al, 2005	Transversal	Brasil	Atraso do desenvolvimento neuropsicomotor	
18	Choice of medical investigations for developmental delay: a questionnaire survey.	Gringras, 1998	Transversal	Estados Unidos	Developmental delay	É um grupo heterogêneo de condições resultantes de consequências de processos genéticos, infecciosos, cromossômicos e uma variedade de outros processos.
19	Genetics and developmental delay.	Mac Millan, 1998	Teórico	Estados Unidos	Developmental delay	
20	Perfil motor em escolares com problemas de aprendizagem.	Rosa-Neto et al, 2005	Transversal	Brasil	Atraso do desenvolvimento neuropsicomotor	É um sintoma de que algo não está de acordo com o esperado.
21	Early intervention in developmental delay.	Kaur et al, 2006	Transversal	Índia	Developmental delay	É o atraso físico, cognitivo, linguagem, social, emocional ou com condição provável de desenvolver atraso.
22	Determinants of developmental delay in infants aged 12 months.	Slykerman et al, 2007	Longitudinal	Nova Zelândia	Developmental delay	É o atraso dos marcos motores que pode não estar, relacionado à déficit cognitivo e sim à hipotonia ou à pobre coordenação motora, sem sinais neurológicos que justifiquem diagnóstico de paralisia cerebral.
23	Avaliação do desenvolvimento neuropsicomotor em crianças de um bairro da periferia de Porto Alegre.	Saccani et al, 2007	Transversal	Brasil	Atraso do desenvolvimento neuropsicomotor	É uma síndrome do desenvolvimento infantil.
24	Caracterização do desempenho motor em escolares com transtorno de déficit de atenção com hiperatividade.	Toniolo et al, 2009	Transversal	Brasil	Atraso do desenvolvimento neuropsicomotor	
25	Developmental delay syndromes: psychometric testing before and after chiropractic treatment of 157 children.	Cuthbert; Barras, 2009	Transversal	Estados Unidos	Developmental delay	
26	Global developmental delay and mental retardation or intellectual disability: conceptualization, evaluation, and etiology.	Shevell, 2008	Teórico	Canadá	Global developmental delay	É um distúrbio ou uma disfunção do desenvolvimento infantil.
27	Facilidades e dificuldades da família no cuidado à criança com paralisia cerebral.	Dantas et al, 2012	Descritivo	Brasil	Atraso do desenvolvimento neuropsicomotor	
28	Global developmental delay – globally helpful?	Willians, 2010	Teórico	Estados Unidos	Global developmental delay	É o atraso em dois ou mais domínios do desenvolvimento, sendo considerado significativo quando ocorre discrepância de 25% ou mais da taxa esperada, ou uma diferença de 1,5 a 2 desvio-padrão da norma em um ou mais domínios em testes norma-referenciados.
29	Present conceptualization of early childhood neurodevelopmental disabilities.	Shevell, 2010	Teórico	Canadá	Global developmental delay	
30	Developmental delay: timely identification and assessment.	Pool et al, 2010	Revisão	Estados Unidos	Developmental delay	
31	Evaluation of Children with Global Developmental Delay: A prospective study at sultan qaboos University Hospital, Oman.	Koul et al, 2012	Transversal	Arábia Saudita	Global developmental delay	
32	Caracterização do perfil diagnóstico e fluxo de um ambulatório de Fonoaudiologia hospitalar na área de linguagem infantil.	Mandrá et al, 2011	Transversal	Brasil	Atraso do desenvolvimento neuropsicomotor	É uma comorbidade do desenvolvimento infantil.
33	Perfil dos pacientes com necessiades especiais de uma clínica de odontopediatria.	Menezes et al, 2011	Transversal	Brasil	Atraso do desenvolvimento neuropsicomotor	É um tipo de necessidade especial do desenvolvimento infantil.
34	Monitoramento do desenvolvimento infantil realizado no Brasil.	Zeppone et al, 2012	Revisão	Brasil	Atraso do desenvolvimento neuropsicomotor	Não aquisição progressiva de capacidades motoras e psicocognitivas que progride no sentido cefalocaudal e proximal-distal.

Após codificação e análise dos artigos, foi construído mapa conceitual segundo as especificações de Novak,^12^ criador dessa ferramenta. O mapa conceitual é considerado um estruturador do conhecimento. Ele pode ser entendido como uma representação visual utilizada para compartilhar significados e se apoia fortemente na teoria da aprendizagem significativa de David Ausubel,^13^ que propõe que o ser humano organiza seu conhecimento por meio da hierarquização dos conceitos.

Existem vários tipos de mapas que podem ser usados em diferentes situações, de acordo com a finalidade específica. O mapa conceitual utilizado na presente revisão é do tipo sistema, que organiza as informações num formato semelhante a um fluxograma e mostra as várias relações entre os conceitos. Para confecção do mapa foi utilizado o *software CmapTools.* 14

## Resultados

Dos 71 artigos selecionados para revisão, 55 (77,5%) eram trabalhos internacionais e 16 (22,5%) nacionais. Os termos *global developmental delay* e *developmental delay* foram os mais frequentes na literatura internacional e surgiram em meados da década de 40 e 60, respectivamente, ganhando força nos anos 90. No Brasil, os termos *retardo do DNPM* e *atraso do DNPM* começaram a serem usados no meio científico a partir da década de 80, mas foi na última década que passaram a ser mais frequentemente citados na literatura.

## Quanto à população enquadrada no termo (tabela 2)

Como citado em 23 artigos, 13 tipos de condições clínicas apresentaram o atraso do desenvolvimento como característica. Na literatura internacional, as primeiras vezes que o termo foi citado foi para se referir a crianças pretermo e aquelas com retardo mental. Posteriormente, outras condições que poderiam apresentar atraso foram englobadas, tais como, paralisia cerebral, autismo, crianças com alterações cromossômicas e com anormalidades congênitas. No final da década de 90, ampliou-se para a população de crianças que não tinham uma patologia de base definida, mas que apresentavam como característica algum tipo de atraso do desenvolvimento.

No Brasil, as primeiras citações do termo também se referiam a crianças com retardo mental e, posteriormente, a crianças com deficiências sensoriais.

## Quanto ao uso do termo (tabela 3)

Foram encontrados 19 artigos que apresentaram sete tipos de situação de aplicação do termo. Internacionalmente, começou a ser usado para crianças com problemas neurológicos que não apresentavam desenvolvimento típico ou, ainda, para aquelas que não atingiam os marcos motores esperados para a idade cronológica. Posteriormente, o conceito de atraso foi operacionalizado por meio de testes de desenvolvimento norma-referenciados. No final da década de 90, o termo passou a ser também usado em crianças menores de cinco anos de idade, enquanto se aguarda diagnóstico definitivo, e também como diagnóstico, porém sem critério específico.

Já no Brasil, o termo começou a ser utilizado na década de 80 como diagnóstico para crianças com retardo mental e, a partir da década de 90, para qualquer criança que apresentasse algum tipo de atraso do desenvolvimento. Só mais recentemente o termo foi utilizado para crianças que apresentam baixa pontuação em testes de desenvolvimento norma-referenciados.

## Quanto à definição do termo (tabela 4)

Trinta e quatro artigos apresentaram definições, nas quais foram identificados 16 conceitos diferentes. Na literatura internacional, as definições do termo começaram a aparecer na década de 90 e incialmente o conceito se referia ao desenvolvimento mais lento que as demais crianças provenientes da mesma cultura, sendo que lentidão no desenvolvimento era atribuída a um grupo heterogêneo de fatores biológicos e ambientais. A partir de 2000, surgiram outras definições do termo baseada nos marcos motores do desenvolvimento, que poderia ser justificada por hipotonia ou pobre coordenação motora, sem causa específica, sendo que o atraso deveria ser quantificado por meio da aplicação de testes de desenvolvimento norma referenciados. Recentemente, o termo foi conceituado pela Academia Americana de Neurologia e o Comitê de Neurologia Infantil^11^ como atraso em dois ou mais domínios do desenvolvimento, sendo considerado significativo quando ocorre discrepância de 25% ou mais da taxa esperada, ou uma diferença de 1,5-2,0 desvios-padrão da norma em um ou mais domínios do desenvolvimento em testes norma-referenciados.

No Brasil, na década de 90 o termo foi conceituado como uma combinação de microcefalia, com anormalidades fonais e desenvolvimento cerebral atípico. Ao longo da última década, ele recebeu várias significações, como sintoma, síndrome, distúrbio, comorbidade e ainda como uma necessidade especial. O artigo brasileiro mais recente define o termo como a não aquisição progressiva de capacidades motoras e psicocognitivas de modo ordenado e sequencial que progride nos sentidos cefalocaudal e de proximal para distal.

A partir da análise das tabelas e das informações obtidas nos artigos, foi construído mapa conceitual (fig. 2), mostrando a origem dos termos relacionados ao atraso do desenvolvimento e evolução ao longo tempo.

**Figura 2:** Mapa conceitual do termo atraso do Desenvolvimento Neuropsicomotor.

## Discussão

A presente revisão mostra que, na literatura internacional, termos relacionados ao *atraso do DNPM* começaram a ser utilizados em estudos sobre o desenvolvimento cognitivo de crianças pretermo. No estudo mais antigo localizado, Benton, 15 em 1940, utilizou o teste de inteligência de Binet para caracterizar o atraso apresentado por crianças nascidas prematuramente. Segundo esse autor, apesar das crianças pretermo não apresentarem inferioridade intelectual, elas sofriam de "inquietação, nervosismo e cansaço", resultando em distração e baixa concentração. Nota-se assim, na literatura científica, que os primeiros trabalhos sobre o atraso usaram testes cognitivos, 16 logo seguido pelo amplo trabalho de Gesell, 17 que ao criar a primeira escala de marcos de desenvolvimento por faixa etária em 1940, deu impulso aos trabalhos subsequentes que procuravam caracterizar atraso do desenvolvimento em diversas populações.

Os trabalhos de Gesell foram difundidos mundialmente, inspirando a construção dos vários testes de desenvolvimento utilizados atualmente e influenciaram o uso do termo atraso do desenvolvimento, tanto que uma das situações de uso mais encontradas no presente estudo foi a operacionalização do termo por meio de testes do desenvolvimento.^18^^,^ 19 ^,^ 20 ^,^ 21 ^and^ 22 Por exemplo, numa das primeiras referências localizadas, Pape e colaboradores^19^ adotaram, em 1978, como critério para atraso do desenvolvimento em bebês, o índice de desenvolvimento abaixo do esperado para a idade avaliado pela primeira versão das Escalas Bayley de Desenvolvimento Infantil,^23^ teste baseado nos trabalhos de Gesell.

No entanto, apesar do termo atraso do desenvolvimento ter surgido na perspectiva neuromaturacional^17^ e ser bastante utilizado na área da saúde da criança, observa-se não ser um termo consensual tanto com relação à população enquadrada no termo (tabela 2),^15^ ^and^ 24 como pelo seu uso (tabela 3).^25^ ^and^ 26 Nas duas situações, o termo é citado de maneira generalista e excessivamente abrangente e esta variedade de possibilidades pode ser justificada pelo método mais usado para identificar crianças com atraso: a triagem do desenvolvimento.^18^^,^ 19 ^,^ 20 ^,^ 21 ^and^ 22

De fato, a triagem do desenvolvimento é a melhor opção para rastrear crianças com problemas do desenvolvimento por ser um procedimento rápido para ser aplicado em populações amplas de crianças de várias faixas etárias.^34^ Porém, alguns trabalhos revisados^5^^,^ 27 ^and^ 28 comentam que avaliações pontuais do desenvolvimento em crianças menores de cinco anos de idade são pouco confiáveis para delimitar o diagnóstico definitivo, sugerindo-se que o termo atraso do desenvolvimento seja usado como diagnóstico temporário.

Um problema é que esse uso, mesmo que temporário, passa a impressão de uma condição relativamente benigna, que se resolve ao longo do tempo. Nno entanto, estudos revisados^29^^,^ 30 ^and^ 31 sobre o desfecho de crianças que apresentaram atraso nos primeiros anos vida mostram persistência de dificuldades do desenvolvimento. Newton e Wraith^32^ afirmam que a maioria das crianças menores de cinco anos de idade com atraso do desenvolvimento irão apresentar algum tipo de dificuldade de aprendizagem na idade escolar, sendo importante investir no diagnóstico correto.

Alguns autores^28^ ^and^ 33 sugerem que, diante desses problemas subsistentes, o acompanhamento do desenvolvimento nesta população seria benéfico. Tal abordagem envolveria reavaliações periódicas em pontos chave do desenvolvimento, com o objetivo não só de identificar os problemas à medida que surgem, com encaminhamento para intervenção precoce, mas também de obter subsídios para a construção do diagnóstico definitivo.^34^ ^and^ 35

Outro aspecto identificado na presente revisão se refere as definições do termo (tabela 4), que somente começaram a surgir em meados da década de 90, porém bastante heterogêneas. Essa necessidade de definir o conceito pode ter sido ocasionada pela falta de padronização, que se torna insustentável com o aumento expressivo de publicações a partir deste período. Alguns autores começaram a conceituar o termo com base nos resultados de seus estudos, como por exemplo, Najman e colaboradores,^36^ em 1992, ao estudar o desenvolvimento de crianças australianas sob a perspectiva da desigualdade socioeconômica, considerada um problema nacional do país, encontraram maior prevalência de atraso do desenvolvimento nas crianças que tinham mães com baixa escolaridade e baixas condições sócio econômicas. Com base neste achado, os autores do estudo conceituaram o atraso como resultado de fatores biológicos e ambientais, dentro de uma cultura específica. Isto é, diferentes fatores interagindo com o desenvolvimento da criança, influenciando na aquisição das habilidades motoras, cognitivas, linguísticas e sociais.

Na última década, em publicações internacionais, observa-se definições mais padronizadas, consistentes com os avanços científicos da área. O conceito mais recente e sugerido pela comunidade médica^10^^,^ 37 ^and^ 38 foi definido operacionalmente pela Academia Americana de Neurologia e pelo Comitê de Neurologia Infantil.^10^ A definição conforme citada anteriormente, estimula o uso de testes validados, com normas e critérios de referência, para subsidiar a mensuração confiável de dados clínicos relevantes que confirmem o atraso do desenvolvimento.^10^ O Comitê inclusive sugere testes que podem ser utilizados para cada domínio: área motora: *Alberta Infant Motor Scale; Peabody Developmental Motor Scale; Bruininks-Oseretsky Test of Motor Proficiency*; área da fala/linguagem: *Language-Peabody Picture Vocabulary Test; Expressive One Word Vocabulary Test; Clinical Linguistic Auditory Milestone Scale; Clinical Evaluation of Language Fundamental;* área do comportamento: *Vineland Adaptive Behavior Scales, Pediatric Evaluation of Disability Inventory, Wee Functional Independence Measure* e para múltiplas áreas: *Batelle Developmental Inventory.* 10

Quanto às definições no Brasil, observa-se, ao longo das décadas, pouca evolução conceitual, mas numerosas significações.^39^^,^ 40 ^,^ 41 ^,^ 42 ^,^ 43 ^,^ 44 ^and^ 45 O conceito implícito no uso do termo também segue a perspectiva neuromaturacional,^17^ porém, não apresenta parâmetros quantitativos, como encontrado no exterior.^10^ Isto é, as definições brasileiras não incentivam o uso de instrumentos de avaliação do desenvolvimento, o que se justifica pelo pequeno número de testes de desenvolvimento infantil com validação e padronização para a população brasileira.

Aliás, no Brasil, a utilização do termo em publicações científicas ocorreu mais recentemente,^46^ em 1982, do que na literatura internacional^15^ e foram encontrados nomes, usos e definições bem particulares. A começar pela terminologia, no Brasil a primeira citação encontrada foi *retardo do desenvolvimento neuropsicomotor* (DNPM), 47 com adição da palavra *neuropsicomotor* que não é usada na literatura internacional. Este termo surgiu em meados da década de 50, usado pelo neurologista Lefévre 48 em sua tese de docência (1950), na qual discute que, para a criança se desenvolver *neuropsicomotoramente*, ela precisa tanto de crescimento e maturação neural como de aspectos psicológicos e motores. Na tese, Lefévre apresenta a primeira escala de avaliação neuromotora para crianças brasileiras, baseada nas obras de Ozeretski (1936) 49 e do psiquiatra Ajuriaguerra (1948), 50 de onde, possivelmente, surgiu a adição do termo *psicomotor*.

O termo mais próximo ao *retardo do DNPM*, encontrado nos trabalhos de língua inglesa revisados, foi *retardo psicomotor* usado por Fenichel (apud Petersen, Kube, Palmer, 1998), 5 para se referir a "crianças que apresentam retardo mental com leve atraso motor, causado por hipotonia leve ou má coordenação motora e não por baixa função cognitiva". É curioso que o termo *neuropsicomotor* seja usado apenas no Brasil. Como o trabalho de Lefévre foi muito influente, isso possivelmente determinou o uso futuro do termo.

Nas primeiras publicações científicas brasileiras, o termo *retardo do DNPM* foi utilizado como diagnóstico para referir crianças com déficit cognitivo e leve atraso motor, sendo bastante utilizado pelos neurologistas do Brasil. Por exemplo, em 1982, Lefévre e Diament, 46 em estudo realizado com o objetivo de mapear os diagnósticos mais comuns em neurologia infantil no Brasil, encontraram que, dentre os 16s diagnósticos mais frequentes, o *retardo do DNPM* foi o terceiro mais comum. Logo em seguida, este termo ficou mais conhecido como *atraso do DNPM*, descrito por Marcondes, 51 mas mantendo a mesma ênfase do uso, como forma de suavizar a terminologia, uma vez que *retardo* associava-se a crianças com comprometimento grave.

No mapa conceitual (fig. 2), observa-se que as definições encontradas são inúmeras, sendo que a literatura internacional, além de apresentar repertório mais rico, tem maior preocupação em uniformizar a definição do termo e incentivo para se pesquisar a causa do atraso, com investimento nos trabalhos mais recentes em exames diagnósticos específicos.^52^ Isso não é observado no Brasil, pois além da literatura sobre o tema ser recente, é escassa, com definições bem particulares e estudos mais voltados para os fatores de risco para atraso.

Em suma, observa-se que o atraso do desenvolvimento é abordado na literatura internacional e nacional sob diversos nomes, diferentes aplicações e conceitos heterogêneos. Entretanto, os estudos chamam a atenção para um fato em comum, de que *algo não está indo bem* com a criança, pois ela não segue a sequência esperada de aquisições importantes para o seu desenvolvimento. Internacionalmente, tem-se observado investimento na padronização da definiçao o que não foi encontrado nas publicações nacionais. 10 ^and^ 11 No exterior, preconiza-se o uso do termo em crianças menores cinco anos de idade que apresentam alterações no desenvolvimento, sempre identificadas por testes padronizados, 5 ^,^ 27 ^and^ 28 sendo recomendadas avaliações periódicas com auxílio de exames complementares ao longo dos primeiros anos de vida, na tentativa de encontrar a causa do atraso e definir o diagnóstico final. 28 ^and^ 53

Definição mais precisa do que se entende por *atraso do DNPM* é condição essencial para a prestação adequada de cuidados. Entretanto, o uso deste termo tem gerado dificuldade para guiar decisões clínicas nos níveis da avaliação, intervenção e definição do prognóstico de crianças pequenas. Internacionalmente, apontam-se caminhos para melhorar a comunicação entre os profissionais da área, com definição padronizada do termo e uso em situações bem específicas. No Brasil, é preciso investir tanto na padronização do uso do termo, como em programas bem documentados de seguimento de crianças com suspeita de atraso. O acompanhamento do desenvolvimento é um processo que pode auxiliar os profissionais e pais a entenderem o que ocorre com a criança, até a delimitação do diagnóstico final, uma vez que o termo *atraso do DNPM* é usado mais adequadamente como diagnóstico temporário.
